# Deep Learning-Based Intelligent Robot in Sentencing

**DOI:** 10.3389/fpsyg.2022.901796

**Published:** 2022-07-18

**Authors:** Xuan Chen

**Affiliations:** Department of Social Work, School of Law and Politics, Zhejiang Sci-Tech University, Hangzhou, China

**Keywords:** deep learning, artificial intelligence, sentencing, law, restricted boltzmann deep learning model

## Abstract

This work aims to explore the application of deep learning-based artificial intelligence technology in sentencing, to promote the reform and innovation of the judicial system. First, the concept and the principles of sentencing are introduced, and the deep learning model of intelligent robot in trials is proposed. According to related concepts, the issues that need to be solved in artificial intelligence sentencing based on deep learning are introduced. The deep learning model is integrated into the intelligent robot system, to assist in the sentencing of cases. Finally, an example is adopted to illustrate the feasibility of the intelligent robot under deep learning in legal sentencing. The results show that the general final trial periods for cases of traffic accidents, copyright information, trademark infringement, copyright protection, and theft are 1,049, 796, 663, 847, and 201 days, respectively; while the final trial period under artificial intelligence evaluation based on the restricted Boltzmann deep learning model is 458, 387, 376, 438, and 247 days, respectively. The accuracy of trials is above 92%, showing a high application value. It can be observed that expect theft cases, the final trial period for others cases has been effectively reduced. The intelligent robot assistance under the restricted Boltzmann deep learning model can shorten the trial period of cases. The deep learning intelligent robot has a certain auxiliary role in legal sentencing, and this outcome provides a theoretical basis for the research of artificial intelligence technology in legal sentencing.

## Introduction

Intelligent technology has been advancing rapidly since the 20th century. With the continuous optimization of human cognition of the world, the ability of intelligent application is also continuously improved. Thus, with the rapid development of artificial intelligence technology (Koos, 2018; Dessi et al., 2020), an intelligent robot has been proposed. With the proposal and development of intelligent robots, this technology has been applied to various fields such as industry, medicine, and agriculture, and has promoted the rapid development of various fields (Yan-Fang and Zhu, 2019; Ting et al., 2019; Zekos, 2021).

At present, although the application of artificial intelligence technology in the fields of industry, medicine, and agriculture has been very mature, its application in the field of law has just started (Guo et al., 2019). Since the 1980s, foreign countries have begun to build artificial intelligence legal systems (Duan et al., 2019). It was not until 2016 that IBM developed an artificial intelligence legal robot that could provide simple legal advice. This was the first application of intelligent robot in the legal field. Subsequently, scholars and companies at home and abroad started to follow IBM’s example and invested a lot of scientific research funds to study the application of intelligent robot technology in the legal field (Yin et al., 2019). At the end of 2016, scientists from several famous foreign universities jointly carried out the application research of intelligent robot technology in the field of law, and successfully used the artificial intelligence program to predict the trial result (Xiong et al., 2020). In recent years, with the rapid development of deep learning, it has not only provided a method to train the network under the structure of the deep neural network but also broke through the cognition of traditional artificial intelligence (Balas et al., 2019). The restricted Boltzmann deep learning model is a generative random two-way connection neural network; the signal is converted between the hidden layer and the visible layer, thus it is widely used. Deep learning seems to make all machine-assisted functions possible, such as driverless cars, preventive medical care, and high-quality movie recommendations (He et al., 2017). Therefore, it is very possible to combine deep learning and artificial intelligence in sentencing with intelligent robot technology (Kumar et al., 2021).

These research results not only make people feel that the application of artificial intelligence technology in the field of law is promising but also play a positive role in the legal trial practice. Based on the success of foreign research results, China began to learn from this technology. Due to the deep-rooted traditional culture and minority culture in China, the legal construction is not perfect, and there are various problems in the trial of criminal cases. For example, in the case of Nie Shubin, who shocked the whole country, Nie Shubin was acquitted of intentionally killing and raping a woman in December 2016, which exposed serious loopholes in the trial of criminal cases in China (Lv et al., 2019). Therefore, under the background of China’s judicial reform, the reform of China’s criminal trial is also on the horizon. The current deep learning, natural language, and other artificial intelligence technologies have been developed quite mature, but the conviction and sentencing of criminal cases are relatively complex, and the theory of artificial intelligence systems is not mature. There are many difficult cases in criminal proceedings that can’t find out the facts, which is not scary in itself, but the judicial thinking and principles of judicial organs are relatively complex. Taking the principle of innocence as an example, when the facts provided by the prosecution are not clear enough to prove that the defendant has a crime, the court should make a judgment that the defendant is innocent. However, intelligent robot technology can’t carry out complex thinking and application of principles, so there is still immaturity. Deep learning algorithms such as decision trees, support vector machines, and shallow neural networks will depend on professionals in the prediction of trials, resulting in poor scalability. However, many researchers are studying the use of deep learning models for case feature extraction and judgment prediction/similar case matching, and have achieved good outcomes. This indicates that intelligent robot technology has good application prospects in judicial adjudication. Artificial intelligence can also simplify the evidence review process, predict trial outcomes, and give people prior legal reference. Therefore, it has been recognized by domestic and oversea experts in the auxiliary sentencing system. The application of intelligent robot in the trial of criminal cases can effectively reduce the misjudgment of cases and promote judicial reform in China.

Thus, the deep learning-based intelligent robot is utilized to assist legal sentencing in this research. As targeted improvements are made according to three issues that need to be solved in the sentencing of intelligent robot technology, it is then applied to legal practice for validation, to analyze and evaluate its effects. It is expected to provide corresponding auxiliary work for legal consultation and trials because artificial intelligence technology can be used (Hess et al., 2020).

## Literature Review

### Research Status

**FIGURE 1:**
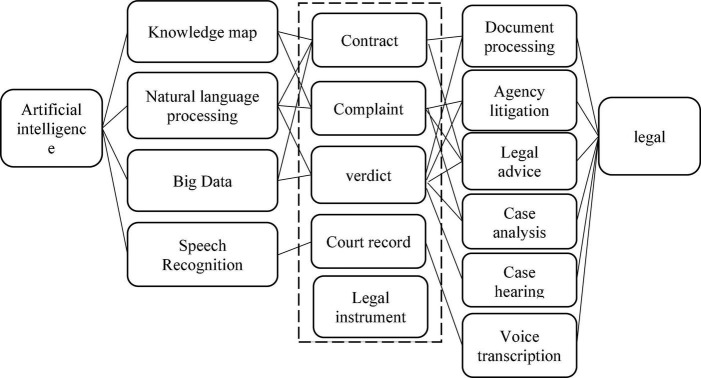
Idea diagram of artificial intelligence technology applied in law.

At present, with the mature development of deep learning, neural network, and other artificial intelligence technologies, the application of intelligent robot technology in the field of law is also the current research boom. The earliest research on the application of artificial intelligence in the field of law began in western countries (Zekos, 2021). In view of the flourishing research on the application of artificial intelligence technology in other fields, in the 1980s, some legal persons with advanced scientific and technological ideas started to build the legal system of artificial intelligence, hoping that the legal system based on artificial intelligence technology could assist lawyers in the trial of some cases (as shown in Figure 1; Lakemeyer, 2018). Some foreign experts established a sentencing model on decision tree algorithm to realize the case classification and prediction of terms of imprisonment sentencing results in judgment direction (Weidong, 2021). Aletras et al. (2017) and other researchers applied the classifier model of N-gram and support vector machine to predict the judgment results of cases, and the average predictive value can reach 79%.

**FIGURE 2:**
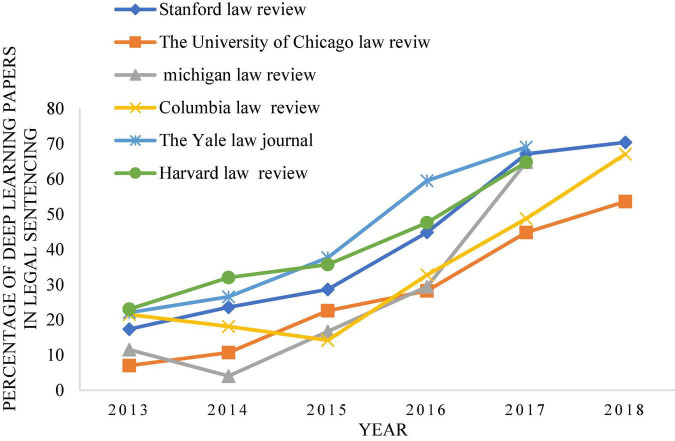
The proportion of papers related to the application of deep learning in the legal sentence in various famous journals in recent years.

**FIGURE 3:**
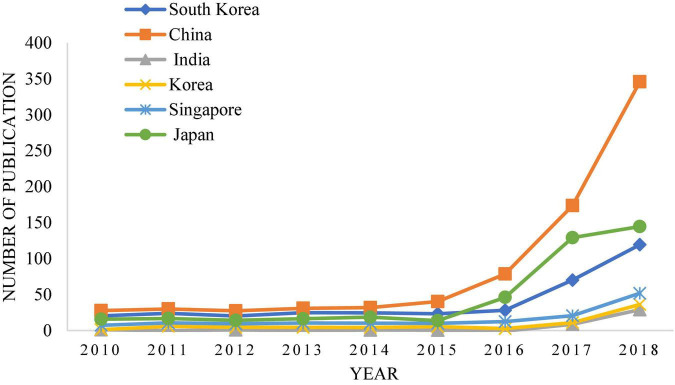
Changes in the application trend of artificial intelligence based on deep learning in the legal field in some Asian countries from 2010 to 2018.

**FIGURE 4:**
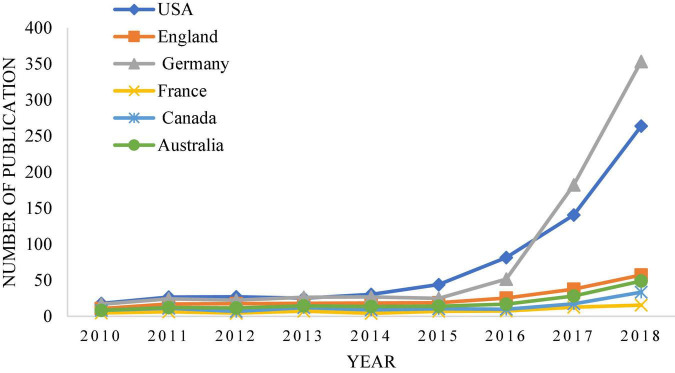
The changing trend of the application of artificial intelligence based on deep learning in the legal field in some western countries from 2010 to 2018.

**FIGURE 5:**
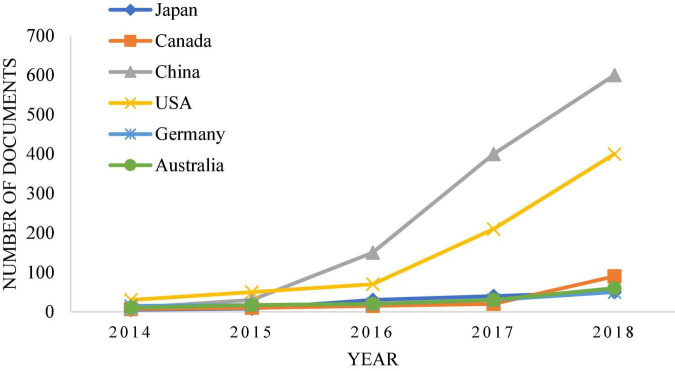
Changes in the published literature on the application of deep learning technology in legal sentencing in some countries from 2014 to 2018.

By contrast, China’s research in this area is relatively late. With the development of technology and times, intelligent robot technology has been widely used now, but the complexity and diversity of legal trials lead to the slow promotion of research on intelligent robot technology in law. Figure 2 shows that the proportion of papers related to deep learning and legal sentencing in major journals has increased significantly over time (Blb et al., 2021). Figures 3, 4 show the growth rate of deep learning in the judicial field in various countries around the world in recent years (Crawford, 2016). Figure 5 shows the changes in the published literature on the application of deep learning technology in legal sentencing in some countries from 2014 to 2018 (Mauer et al., 2021). Although the research in this field is carried out relatively late in China, the development speed is quite fast in recent years.

### Research Results at Home and Abroad

**FIGURE 6:**
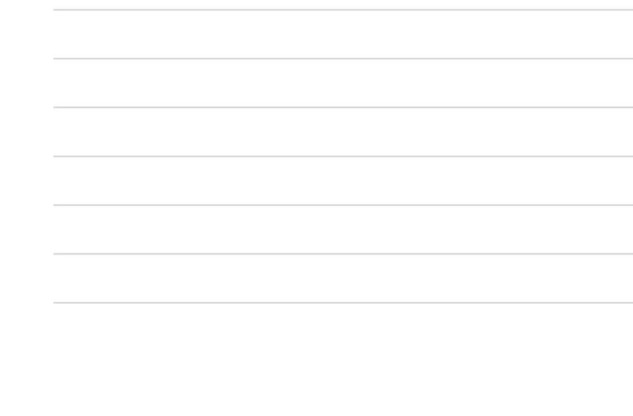
The retrial initiation rate and judgment alteration rate of the intermediate people’s court of Guangdong province.

**FIGURE 7:**
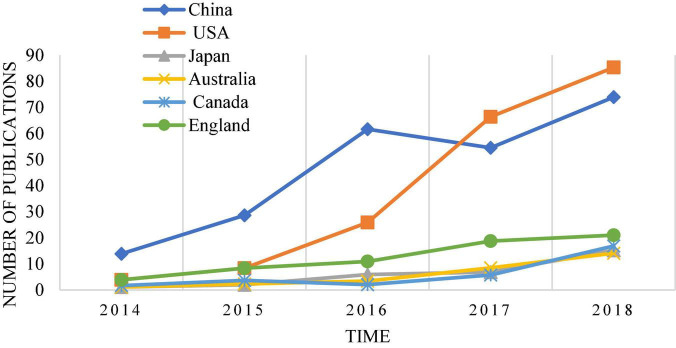
The trend of the combination of artificial intelligence and law in various countries over time.

Since the 1980s, the application research of artificial intelligence technology in the field of law has been increasing, and the research abroad is earlier than that in China. For example, Carrel (2018) pointed out in his research that with the increase in the use of artificial intelligence and machine learning, the nature of legal services has undergone great changes. People gradually realized that legal education and service mode needs to build data and technology platform, hoping to provide customers with the best service to meet their expectations with the help of technology. Mireile and Reynoldson (2017) pointed out that the current wave of artificial intelligence determined the popularity of intelligent robot in the field of law, and the establishment of a legal service system based on artificial intelligence needed to be based on machine experience. In this study, the hypothesis of law was compared with the hypothesis of a computing system. It is discovered that intelligent robot systems generated from large amounts of empirical data were more accurate and successful in predicting the content of empirical laws. In the study of Kingston (2019), taking “what laws apply if a self-driving car kills a pedestrian” as an example, the need to establish an artificial intelligence legal service system to solve the problem of legal liability was analyzed. This research discusses whether the intelligent robot system is applicable to explore criminal liability. Hu (2016) quantified the degree of social harm and personal danger of crime from mathematical principles in their study, which promoted the application of artificial intelligence in sentencing. Yang et al. (2018) believed that although sentencing decisions were affected by more than 200 factors, sentencing laws and practices seemed to make decisions automatically. Therefore, deep learning is adopted to develop and test the intelligent sentencing algorithm and is taken as an auxiliary means of the existing sentencing practice. The results indicated that the widespread adoption of artificial intelligence computer-assisted judgment should be considered to be adopted in people’s life. Chalkidis and Dimitrios (2019) applied deep learning to legal analysis, and the word2vec model was adopted to share pre-trained legal words on a large corpus, which solved the challenging legal language processing problem. In Guangdong province, it developed the “Trial–centric Litigation Service Software” system developed by a high court in Shanghai, which greatly improved the retrial initiation rate and judgment alteration rate of the intermediate people’s court, as shown in Figure 6 (Ribeiro et al., 2018). Figure 7 shows the changes and startups in research on the adoption of artificial intelligence for legal smart sentencing in some countries around the world over time, both of which show an upward trend over time, and the research volume in the United States and China has increased rapidly (Li, 2019).

To sum up, at present, there are relatively few research on the application of intelligent robot system in legal sentencing. From the beginning of proposing the combination of artificial intelligence and law to now, the progress of relevant research is relatively slow, which may be related to the complexity and diversity of legal events. However, it has also achieved some success. Therefore, in the text, the study of intelligent robot based on deep learning technology in the field of legal intelligence sentencing is carried out (Sun et al., 2018; Munir et al., 2019).

## Introduction of Related Concepts

### Sentencing

Col sentencing, also known as penalty discretion, refer to the criminal justice activities in which the people’s court determines whether the offender is sentenced to a penalty, what kind of punishment, and whether the punishment should be immediately executed based on the determination of the crime in accordance with the provisions of the criminal law (Bainbridge and Webb, 2017; Nemitz, 2018). In this research, the characteristics of sentencing were summarized.

First, the constitution is the fundamental law of our country. According to the constitution, only the people’s courts, not other organs and institutions of the state, can exercise criminal jurisdiction. It is one of the components of criminal jurisdiction and one of the components of national penal power.

Second, general trial activities are classified into criminal trial activities, civil trial activities, and administrative trial activities. Sentencing mainly involves the issue of punishment. It is a criminal trial activity, and the trial results are relatively serious.

Third, sentencing is based on a criminal conviction. There is no sentencing without conviction.

Fourth, the content of sentencing generally refers to the determination of issues related to punishment.

The task of sentencing refers to the problem that sentencing needs to solve. The problems solved mainly include the following four aspects (Etzioni and Etzioni, 2017). First, sentencing involves deciding whether to impose a sentence on an offender. As we all know, the basic condition for sentencing is guilt, but it does not mean that the offender will be subject to criminal sanctions. According to the provisions of the criminal law of our country, people who meet the requirements of guilt in some cases are exempted from punishment by the criminal law. Second, based on criminal punishment, the measurement of punishment needs to determine the specific way and degree of punishment. Third, the sentencing also needs to decide whether the offender should be sentenced immediately. In general, sentences imposed on criminals for legitimate and valid criminal offenses need to be carried out immediately. However, the probation system is an exception. For some criminals, punishment is suspended if they meet the conditions stipulated by the law. Fourth, sentences need to decide how to punish when combined penalties are used. In judicial practice, sentencing is faced with more complicated situations, sometimes for the punishment of one person and one crime, and sometimes for some crimes. In the case of a person committing a crime, a certain amount of punishment is required for the crime, and the punishment shall be carried out in accordance with the principle of combined punishment (Allen et al., 2019).

### Principle of Sentencing

Sentencing is a part of the criminal trial, which is very important in the judicial process (Liew, 2018). The principle of sentencing is used to guide them to make a good judgment in the judicial process and avoid sentencing deviation. According to article 61 of China’s criminal law, there are two principles for sentencing: one is based on the criminal facts and the other is based on the criminal law (Branting, 2017).

The principle based on criminal facts contains four parts. First, it is necessary to find out the facts of the crime with an objective and fair attitude, and deal with the facts of the crime impartially and proactively. The first step of the trial of criminal cases is to earnestly implement laws and regulations. Second, when determining the nature of a crime, the basis is the provisions of the criminal law and the conditions of the crime, which requires a correct conviction, not the experience of the supervisor. Third, it is necessary to comprehensively grasp the circumstances of the crime. The nature of the crime and the circumstances of the crime are factors that measure the degree of danger in the criminal society. For example, under the same nature of the crime, crime circumstances determine the degrees of social harm. Therefore, criminal circumstances have a good guiding role in sentencing. Fourth, the degree of social harm by crime is determined according to various factors (Cath et al., 2018).

Take criminal law as the criterion. The principle of criminal law, which takes criminal law as the criterion, is also the basic requirement to realize sentencing. It consists of four parts: first, the type of punishment and term of imprisonment shall be subject to the general provisions of the criminal law and shall not exceed the scope prescribed by the criminal law. At the same time, China’s criminal law has stipulated the general conditions for the application of the penalty. When sentencing, neither the lower limit of legal time limit nor the upper limit of legal time limit can be exceeded. Second, all kinds of punishment methods can only be applied according to the general conditions and scope of the criminal law. For example, according to the general provisions of the criminal law, the death penalty is only applicable to criminals who commit extremely serious crimes (Jayakumar et al., 2019).

## Problems That Artificial Intelligence Needs to Solve in Sentencing Based on Deep Learning Technology

### The Conversion of the Remaining Constitution of the Crime Fact for Conviction

In criminal cases, to accurately grasp the conviction of the facts of the case, it is necessary to distinguish between the judgment and sentencing circumstances. According to the characteristics of a criminal trial in our country, the most basic elements in the whole process of a criminal trial are the circumstances of conviction and sentencing. All the subjective and objective facts related to crime are called criminal facts. Therefore, the criminal facts are classified into the criminal constitute facts and non-criminal constitute facts. But in the whole process of sentencing, it is necessary to investigate the facts of the non-criminal constitution.

**FIGURE 8:**
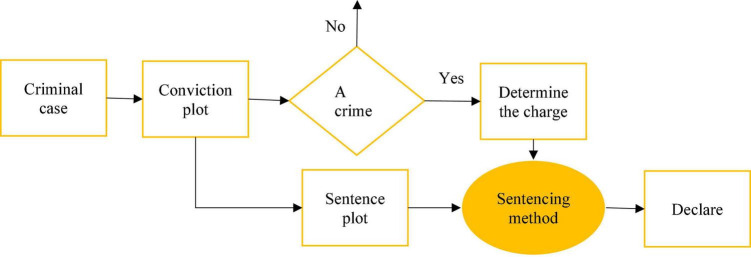
The conversion of the remaining constitution of the crime fact for conviction.

Through the research and analysis of the provisions of the criminal law and the theory of crime constitution in China, some charges in the criminal law contain some selective elements in the crime constitution elements, and the conviction does not need to include all the elements. At this point, the subjective and objective facts covered by the constitution of the crime outweigh the necessity of conviction. Therefore, judging the facts that meet the minimum requirements for the constitution of a crime becomes a mark to distinguish the circumstances of conviction from the circumstances of sentencing (Business et al., 2018). In the design of the artificial intelligence concept based on deep learning technology, it is difficult to distinguish the crime constitution facts from the non-crime constitution facts through the understanding of the crime facts and convert the remaining crime constitution facts into sentencing circumstances, to accurately measure and punish, as shown in Figure 8.

### Insufficient Development of Data Resources Based on Artificial Intelligence in the Legal Field

**FIGURE 9:**
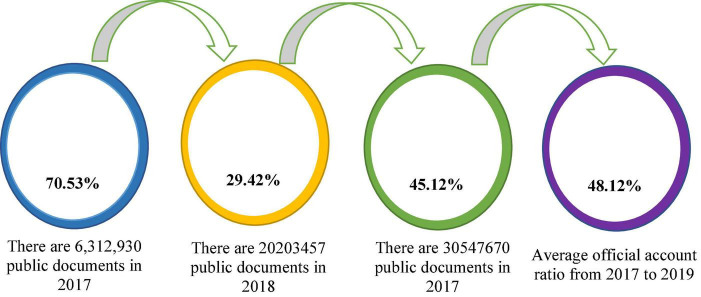
Proportion of the number of open cases on China Judgment Document Network from 2017 to 2019.

Although the current Internet technology has been very mature, the current upload rate on the judicial database is very low, and the data comes from the judicial website familiar to the public, for example, judicial data such as the China Judgment Document Network and the China Trial Process Information Open Network. According to these uploaded data, the uploading of judicial data is not optimistic and the upload rate is less than 50% of the actual settlement. Figure 9 shows the proportion of the number of open cases on the China Judgment Document Network from 2017 to 2019 (He, 2019).

### Optimization of the Trial Cycle in Sentencing

**FIGURE 10:**
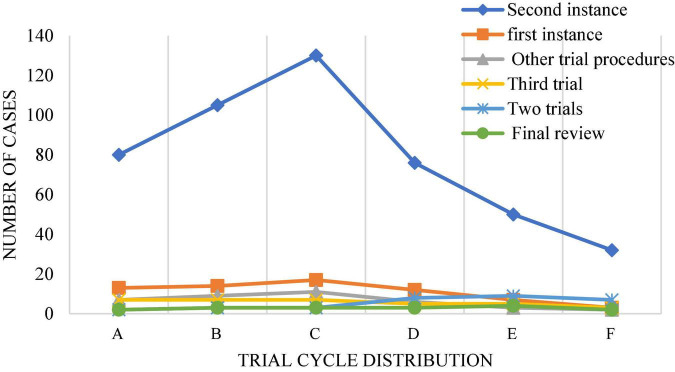
Case trial cycle. (A: <= 2 months; B: 2–4 months; C: 4∼6 months; D: 6∼8 months; E: 8∼10 months; F: > 10 months).

**FIGURE 11:**
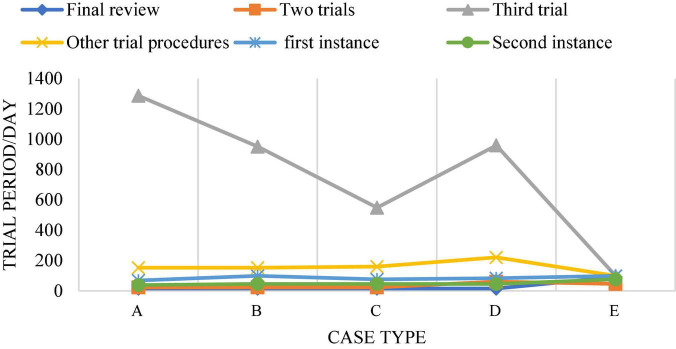
Distribution of trial cycles of different types of cases. (A: Intellectual property plagiarism, infringement dispute; B: other intellectual property competition disputes; C: intellectual property contract dispute; D: unfair competition dispute; E: monopoly dispute).

In China’s judicial practice, sentencing circumstances are mostly defined as sentencing circumstances of law, sentencing circumstances of judicial interpretation, and discretionary sentencing circumstances. The law gives the judge a certain discretion in the trial of the case. In the establishment of an artificial intelligence system, it is usually a complex process for machines to form values consistent with human beings, which is the difficulty of establishing artificial intelligence system based on deep learning. When applying artificial intelligence system to sentencing, the lawyer’s value reasoning process needs to be stimulated by the correct value reasoning method. In this process, the sentencing time will be extended, and the trial period of cases and procedures at the present stage is also a problem that needs to be solved in the application of artificial intelligence in the field of law. Figures 10, 11 show the current trial cycle of various stages (Stockholm, 2021). The trial cycle of most cases needs to be more than 10 months, which is particularly long. If this problem is solved, it will greatly promote the development of the judicial field.

## Countermeasures and Application of Artificial Intelligence Based on Deep Learning in Legal Sentencing

### Knowledge Generation Application of Intelligent Robot System Under Deep Learning in the Legal Field

Although intelligent robot system has a good role in assisting sentencing currently, especially when combined with big data, it can improve judicial efficiency and achieve justice in the judicial process to a certain extent. However, long-term criminal sentencing through intelligent robot will cause the court or judge’s jurisdiction to be impacted, justice will also be challenged, and there may be implicit discrimination against the interests of marginalized groups. Therefore, intelligent robot sentencing must be an assistance in the sentencing of criminals based on defending the dominance of judges, to ensure the benign operation and orderly development of sentencing. In artificial intelligence sentencing cases, it is necessary to accurately identify the remaining criminal facts and convert them into sentencing circumstances. Then, the statutory sentencing circumstances and discretionary sentencing circumstances are dealt with separately. The judge needs to conduct value reasoning on the sentencing results of the quantitative criminal emotion and discretionary criminal emotion, to get the possible impact on the sentencing and publish the results after comprehensive consideration.

When adopting deep learning technology to generate legal knowledge, it is necessary to describe relevant legal knowledge and concepts as well as criminal facts. When judging the constitution of a crime fact or the constitution of a non-crime fact, deep learning technology is first adopted to train the learning network strategy by taking the existing classic judgment data of the court as training samples. Then, the model is used to generate the corresponding legal sentencing knowledge and form the knowledge base belonging to the model (Camps et al., 2018; Harrou et al., 2020; Hashimoto et al., 2020).

### Knowledge-Activated Reasoning of Intelligent Robot System Under Deep Learning in the Legal Field

To activate the knowledge base of the deep learning model, an algorithm is required to search the game tree, which is a supervised learning network and an augmented learning network provided by the model. According to the study and self-study of classic cases, the knowledge base is constantly updated. Supervised learning network and reinforcement learning network are the deepening of multi-layer neural network or convolutional neural network. Through convolution operation, the depth of figure extraction is continuously deepened. The whole training process of the neural network automatically modulates the parameters of convolutional kernel, and further produces classification features without supervision (Wang et al., 2019). Therefore, this process can activate the knowledge base to complete the classification of sentencing circumstances. In this research, the restricted Boltzmann deep learning model is utilized for auxiliary sentencing. The restricted Boltzmann network is a neural network that generates random bidirectional connections, in which the signal is converted between the hidden layer and the visible layer. Thus, it is widely applied.

### Research Process

**FIGURE 12:**
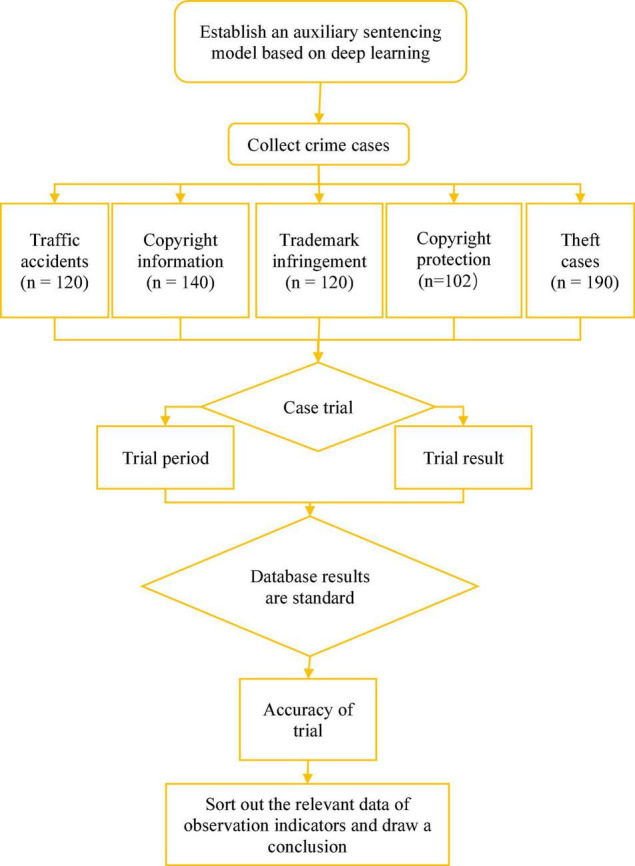
Schematic diagram of the research process.

The flow chart of this research process is displayed in Figure 12. First, it is necessary to establish an auxiliary sentencing model under deep learning, and second, data for different types of cases are collected. After, the model is applied for the trial of the case, to obtain the trial period and trial results. With the database results as the standard, the accuracy of its trials is assessed.

(1) *Data sources and analysis methods*

The dataset adopted is provided by the National Crime Information Center (NCIC) database (Shi, 2021). It is used to select cases for empirical analysis in which different types of crimes were sentenced to fixed-term imprisonment in 2019–2020. Among these crimes, there are 120 traffic accidents, 140 copyright information cases, 120 trademark infringements, 102 copyright protection cases, and 190 thefts. The sentencing technology-assisted trials based on the deep learning model are constructed, and the trial period of the cases and the accuracy of trials (subject to the database results) are taken as the evaluation standards of the application value. For the trial period, the time used for the first trial, the second trial, the two trials, the third trial, other trial procedures, and the final review is analyzed and compared (Hooshmand et al., 2020).

(2) *Computer-assisted sentencing*

The specific steps to establish computer-assisted sentencing are as follows. First, an in-depth investigation is conducted on the sentencing situation of the case and the sentencing experience of trial experts, several representative judgments approved by experts are collected, and the sentencing elements are extracted and quantified from these verdicts. Artificial intelligence based on the restricted Boltzmann deep learning model is adopted to evaluate the circumstance for sentencing. By training classic cases, extracting and quantifying similar plots from new cases and substituting them into the sentencing model of deep learning, the declaratory punishment of this case is obtained.

(3) *Deep learning model*

**FIGURE 13:**
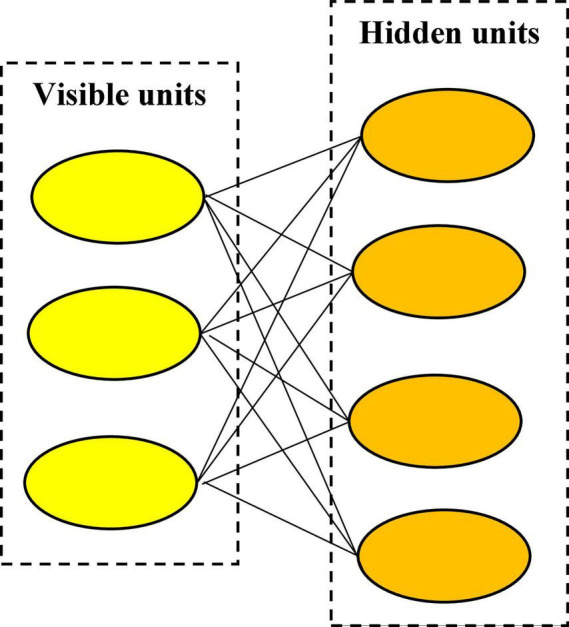
Model structure of the restricted Boltzmann machine.

Hiton and Uebergang (2017) built the neural network model by adding random mechanisms into the neural network model. There are two main differences between random networks and other neural networks. (I) In the learning phase, the random network does not adjust the weight based on a certain deterministic algorithm like other networks but is modified according to a certain probability distribution. (II) In the operation stage, the random network does not perform state evolution according to a certain deterministic network equation but determines its state transition according to a certain probability distribution. The net input of a neuron can’t determine whether its state is 1 or 0, but it can determine the probability of its state is 1 or 0. Various network models are randomly generated. This network model is called Boltzmann deep learning model. It can learn data without supervision. Figure 13 is a schematic diagram of the network structure of the restricted Boltzmann deep learning model (Liu and Yan, 2020). Figure 13 shows that the restricted Boltzmann deep learning model is composed of two layers of neural networks: the visible layer and the hidden layer. The nodes in the network structure of restricted Boltzmann deep learning models are binary variable nodes. And the nodes of the visible and hidden layers are not connected.

If the visible layer is set as *v* and the hidden layer is set as *h*, then the probability *P*(*h*|*v*) of Boltzmann distribution satisfied by the visible layer is expressed as equation 1.


(1) $$P\left(h|v\right)=\prod_{j}^{n}P\left(h_{i}|v\right)$$

In the neural network, the joint probability distribution of visible and hidden nodes is expressed by equation (2):


(2) $$P_{\theta }\left(v,h\right)=\frac{1}{Z\left(\theta \right)}e^{-E\left(v,h,\theta \right)}=\frac{1}{Z\left(\theta \right)}\prod_{i,j}e^{W_{ij}v_{i}h_{j}}\prod_{i}e^{b_{i}v_{i}}\prod_{j}e^{a_{j}h_{j}}$$

Among them, *Z*(θ) = ∑_*v*,*h*_ *e*^−*E*(*v*,*h*,θ)^.

The RBM energy function is as follows:


(3) $$\begin{array}{c} E\left(v,h,\theta \right)=-a^{T}{\text{h-b}}^{T}{\text{v-v}}^{T}wh\\ \;=-\sum_{i,j}w_{ij}v_{ij}h_{j}-\sum_{i}b_{i}v_{i}-\sum_{J}a_{j}h_{j} \end{array}$$

Among them, *E* is the expected value; θ = {*W*,*a*,*b*} is the model parameter; *a*_*j*_ is the deviation of the input variable *v*_*i*_; *b*_*i*_ is the deviation of hidden variable *h*_*j*_, *W*_*ij*_ is the weight of the interaction between *i* and *j* in the visible layer and the hidden layer. The definition of marginal probability distribution *P* of the data vector *v* is expressed by equation (4).


(4) $$\begin{array}{cc} P\left(v;\theta \right)=\sum_{h}\frac{e^{-E\left(v,h,\theta \right)}}{\sum_{v,h}e^{-E\left(v,h,\theta \right)}}=\frac{1}{Z\left(\theta \right)}\sum_{h}e^{-E\left(v,h,\theta \right)} & \\ \;=\frac{1}{Z\left(\theta \right)}e^{b^{T}\text{v}}\sum_{h}e^{{}^{a^{T}h+b^{T}v+v^{T}wh}} & \\ \;=\frac{1}{Z\left(\theta \right)}e^{b^{T}\text{v}}\prod_{j=1}^{F}\sum_{h_{j}\in \left\{0,1\right\}}e^{\left(ajhj+\sum_{i=1}^{D}W_{ij}v_{i}h_{j}\right)} & \\ \;=\frac{1}{Z\left(\theta \right)}e^{b^{T}\text{v}}\prod_{j=1}^{F}\left(1+e^{\left(a_{j}h_{j}+\sum_{i=1}^{D}W_{ij}v_{i}h_{j}\right)}\right) & \end{array}$$

Among them, *Z*(θ) represents the normalization factor, *T* represents kinetic energy, and *F* represents constant.

Equation (4) is the marginal distribution function of visible units. The characteristic of this function is that when the energy of the system changes, it gives a high probability to the system with low energy and a low probability to the system with high energy. Therefore, according to the energy function, the probability of equations (5) to (9) is defined as follows.


(5) $$P\left(v|h;\theta \right)=\prod_{i}P\left(v_{i}|h\right)$$

(6) $$P\left(v_{i}=1|h\right)=\varphi \left(b_{j}+\sum_{j}h_{j}W_{ij}\right)$$

(7) $$P\left(h|v;\theta \right)=\prod_{j}P\left(h_{j};v\right)$$

(8) $$P\left(h_{j}|v\right)=\varphi \left(a_{j}+\sum_{i}v_{i}W_{ij}\right)$$

(9) $$\varphi \left(x\right)=\frac{1}{1+{\text{e}}^{-x}}$$

In equations (5) to (9), θ is the parameter of the restricted Boltzmann deep learning mode; *b* and *a* are the biases of the visible unit and the hidden unit, respectively; *x* represents the learning rate, *v* represents the visible variable *v*, and *h* represents hidden variables.

The parameters in the experiment are set. The learning rate (lr) is [0.01, 0.001, 0.0001] and the batch size is [16, 32, 64, 128]. The optimizer adopts the Adam optimizer, the number of iterations is 30, and the activation function is the Relu activation function. The research is carried out according to the above parameters, to compare the effect of algorithm assistance (Yuan and Wu, 2020).

(4) *Result verification and analysis*

**FIGURE 14:**
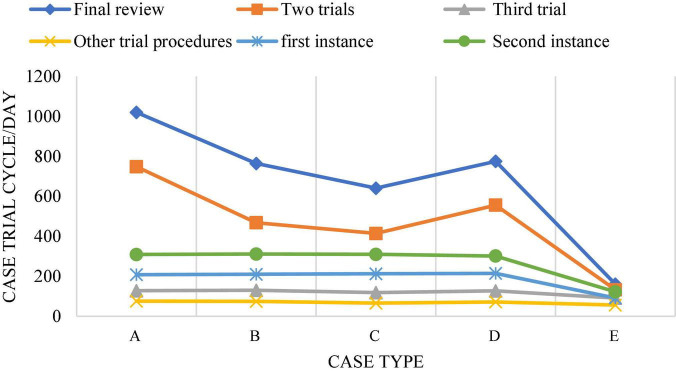
The trial period of different types of cases under normal circumstances. (A: Traffic accident; B: copyright infringement; C: trademark infringement; D: copyright protection; E: theft infringement).

**FIGURE 15:**
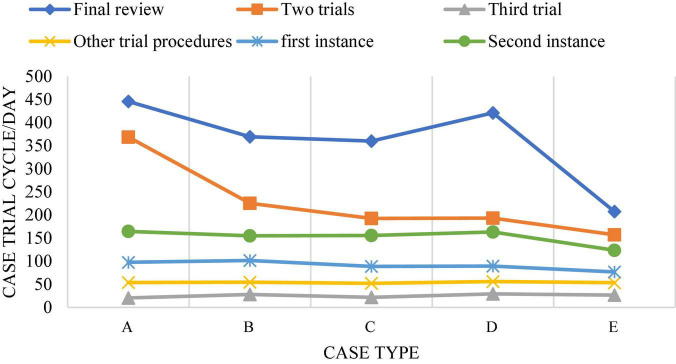
The trial cycle of different types of cases with the help of artificial intelligence based on deep learning technology. (A: Traffic accident infringement; B: copyright infringement; C: trademark infringement; D: copyright protection; E: theft infringement).

**FIGURE 16:**
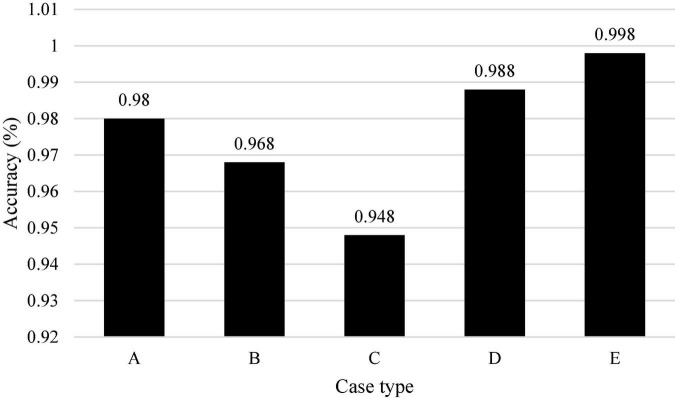
The accuracy of the trial results. (A: Traffic accident infringement; B: copyright infringement; C: trademark infringement; D: copyright protection; E: theft infringement).

Figure 14 shows the trial cycle distribution diagram of each case, and Figure 15 shows the cycle distribution diagram after the adoption of legal sentencing based on artificial intelligence technology. Figures 13, 14 show that artificial intelligence based on deep learning technology greatly shortens the trial period of cases when sentencing. Under normal circumstances, the final trial periods for traffic accidents, copyright information, trademark infringement, copyright protection, and theft cases are 1,049, 796, 663, 847, and 201 days, respectively. The final trial periods for traffic accidents, copyright information, trademark infringement, copyright protection, and theft cases *via* artificial intelligence evaluation based on the restricted Boltzmann deep learning model are 458, 387, 376, 438, and 247 days, respectively. This is similar to the research results of Nweke et al. (2018), showing that artificial intelligence based on the restricted Boltzmann deep learning model greatly shortens the trial period of different types of cases. Surprisingly, the final trial period for theft cases is 201 days under normal circumstances, while the final trial period for theft cases under artificial intelligence is 247 days. It may be because most of the theft cases involve a small amount, and the case is relatively simple and clear, so artificial judgment is more intuitive and faster. Figure 16 represents the accuracy of the trial results under the deep learning algorithm-based sentencing technology. The 117 cases of traffic accident, 135 cases of copyright infringement, 113 cases of trademark infringement, 100 cases of copyright protection, and 189 cases of theft are trialed correctly. The accuracy is more than 92%, which is pretty high; and the technology is of a high application value.

## Conclusion

To analyze the application of intelligent robot system under deep learning technology in the sentencing field, in this study, the basic concept of sentencing, sentencing principles, and the restricted Boltzmann deep learning model were introduced, and three questions through the combination of deep learning technology model and law were proposed. That is, the conversion of the remaining constitution of the crime fact for conviction, the insufficient development of artificial intelligence data resources in the legal field, and the optimization of the trial cycle in sentencing. The above three questions are the application difficulties of deep learning technology in legal sentencing. Finally, based on the example, the application of intelligent robot system based on deep learning in legal sentencing was verified. The verification results showed that it was feasible to verify the criminal facts, which indicated that the intelligent robot system under deep learning technology could help lawyers to judge cases in the field of legal sentencing.

The validity of legal intelligence sentencing is analyzed by adopting intelligent robot system based on the restricted Boltzmann deep learning model. However, the deep learning model adopted in this research is relatively basic, and the optimization analysis of the restricted Boltzmann deep learning model will be considered. Moreover, the empirical part only shows the quantitative data results of the trial period of the case, which is insufficient to solve other problems. In the future, it should increase the selection and sorting of cases and strive to apply deep learning artificial intelligence to more legal issues. In conclusion, the results can provide ideas for the further study of the legal intelligent auxiliary system and promote the scientific and technological reform and innovation of the judiciary.

## Data Availability Statement

The raw data supporting the conclusions of this article will be made available by the authors, without undue reservation.

## Author Contributions

The author confirms being the sole contributor of this work and has approved it for publication.

## Conflict of Interest

The author declares that the research was conducted in the absence of any commercial or financial relationships that could be construed as a potential conflict of interest.

## Publisher’s Note

All claims expressed in this article are solely those of the authors and do not necessarily represent those of their affiliated organizations, or those of the publisher, the editors and the reviewers. Any product that may be evaluated in this article, or claim that may be made by its manufacturer, is not guaranteed or endorsed by the publisher.
